# Structural Musculotendinous Parameters That Predict Failed Tendon Healing After Rotator Cuff Repair

**DOI:** 10.1177/23259671231196875

**Published:** 2023-09-19

**Authors:** Maurits G.L. Olthof, Martin Flück, Paul Borbas, Paola Valdivieso, Marco Toigo, Fabian Egli, Jethin Joshy, Lukas Filli, Jess G. Snedeker, Christian Gerber, Karl Wieser

**Affiliations:** † Department of Orthopedics, Balgrist University Hospital, University of Zurich, Zurich, Switzerland; ‡ Laboratory for Muscle Plasticity, Department of Orthopedics, University of Zurich, Zurich, Switzerland; § Institute for Biomechanics, ETH Zurich, Zurich, Switzerland; ‖ Department of Radiology, Balgrist University Hospital, University of Zurich, Zurich, Switzerland; Investigation performed at Balgrist University Hospital, Zurich, Switzerland

**Keywords:** muscle fatty infiltration, muscle atrophy, muscle fiber type, rotator cuff reconstruction, prospective cohort study, predictive model

## Abstract

**Background:**

Healing of the rotator cuff after repair constitutes a major clinical challenge with reported high failure rates. Identifying structural musculotendinous predictors for failed rotator cuff repair could enable improved diagnosis and management of patients with rotator cuff disease.

**Purpose:**

To investigate structural predictors of the musculotendinous unit for failed tendon healing after rotator cuff repair.

**Study Design:**

Cohort study; Level of evidence, 2.

**Methods:**

Included were 116 shoulders of 115 consecutive patients with supraspinatus (SSP) tear documented on magnetic resonance imaging (MRI) who were treated with an arthroscopic rotator cuff repair. Preoperative assessment included standardized clinical and imaging (MRI) examinations. Intraoperatively, biopsies of the joint capsule, the SSP tendon, and muscle were harvested for histological assessment. At 3 and 12 months postoperatively, patients were re-examined clinically and with MRI. Structural and clinical predictors of healing were evaluated using logistic and linear regression models.

**Results:**

Structural failure of tendon repair, which was significantly associated with poorer clinical outcome, was associated with older age (β = 1.12; 95% CI, 1.03 to 1.26; *P* = .03), shorter SSP tendon length (β = 0.89; 95% CI, 0.8 to 0.98; *P* = .02), and increased proportion of slow myosin heavy chain (MHC)–I/fast MHC-II hybrid muscle fibers (β = 1.23; 95% CI, 1.07 to 1.42; *P* = .004). Primary clinical outcome (12-month postoperative Constant score) was significantly less favorable for shoulders with fatty infiltration of the infraspinatus muscle (β = –4.71; 95% CI, –9.30 to –0.12; *P* = .044). Conversely, a high content of fast MHC-II muscle fibers (β = 0.24; 95% CI, 0.026 to 0.44; *P* = .028) was associated with better clinical outcome.

**Conclusion:**

Both decreased tendon length and increased hybrid muscle fiber type were independent predictors for retear. Clinical outcome was compromised by tendon retearing and increased fatty infiltration of the infraspinatus muscle. A high content of fast MHC-II SSP muscle fibers was associated with a better clinical outcome.

**Registration:**

NCT02123784 (ClinicalTrials.govidentifier).

Rotator cuff tears are frequent and constitute the most common cause of shoulder dysfunction and pain.^53^ Surgical reconstruction of rotator cuff tears has shown reliable improvements in pain and function. However, healing after rotator cuff repair remains a major clinical challenge with reported failure rates of 13% to 94%.^18^ These failures are associated with inferior clinical outcomes and progression of musculotendinous degenerative changes.^4,14,52^ Despite ongoing advances in surgical treatment and fixation strength, rehabilitation, and patient education, the failure rate remains high, and there may be limitations in intrinsic healing potential of some rotator cuff tears.

Chronic retraction of the musculotendinous unit is associated with structural muscular changes, including muscle atrophy and fatty infiltration, which have been shown to be independent predictors for failure of rotator cuff repair.^31,36^ As the sensitivity of the different muscle fiber subtypes (slow myosin heavy chain [MHC]–I, fast fibers [MHC-IIa, MHC-IIx, MHC-IIb], and hybrid type [slow MHC-I/fast MHC-II]) to atrophy signals is dependent on physiological and metabolic stimuli, muscle atrophy is associated with change in muscle fiber type composition.^51^ As loss of elasticity of the musculotendinous unit is associated with more difficult anatomical reduction to the insertion site and increased tension of the repair,^29^ we hypothesized that muscle with increased proportion of hybrid-type muscle fibers is more prone to failure of rotator cuff repair.

In addition to structural changes of the muscle, structural changes of the tendon – including tear size and tendon length – have been associated with failure of rotator cuff repair.^40^ Previous research indicated that retraction of the musculotendinous unit involved nonsynchronous shortening of the tendon and muscle.^38^ Furthermore, the combination of tendon shortening and fatty infiltration of the muscle substantially improved the preoperative predictive value for rotator cuff reconstruction failure.^40^ However, the evidence is still limited, and it remains unconfirmed that tendon length is an independent predictor for retear.

Despite previous research efforts, systematic multivariate analyses of factors associated with repair failure are sparse. In a meta-analysis that included 108 studies with evidence levels from 1 to 4, fatty infiltration, tear size, advanced age, and double-row repair were found to be associated with morphological retear.^36^ Although these studies indicate a strong influence of intrinsic parameters of the musculotendinous unit, the validity of the measurement methods can be improved. Most studies assess fatty infiltration based on magnetic resonance imaging (MRI) adaptation of the Goutallier classification.^17,23^ However, since this classification is influenced by retraction of the musculotendinous unit^38^ and has not shown a better than moderate interrater reliability,^17,30,38^ it may not represent an accurate quantification of muscular fatty infiltration. Advances in MRI technology allow us to reliably quantify muscle fat fraction using Dixon sequences.^10,24,52^

In the present study, we investigated the histological and radiographic musculotendinous predictors for retear after rotator cuff repair using Dixon MRI and histology of intraoperative muscle biopsies. We hypothesized that muscle fiber type composition would be significantly associated with failure of rotator cuff repair.

## Methods

### Research Design

Between May 2014 and March 2017, a total of 115 patients (116 shoulders) were enrolled in this institutional review board–approved study. Since the homogeneity of the study population could not be predicted, the study size was based on a previous study of 84 patients that investigated the histopathological parameters of the rotator cuff.^6^ Included in that study were consecutive patients who were undergoing arthroscopic rotator cuff repair at a single institution. All patients had a full-thickness supraspinatus (SSP) tear with fatty infiltration of the SSP muscle of not more than Goutallier stage 2. Excluded were patients with previous surgery on the shoulder, osteoarthritis of the glenohumeral joint, inflammatory joint diseases, or use of oral steroids or immunosuppressive drugs. All included patients provided written informed consent.

### Surgical Procedure

Tendon, capsular, muscular, synovial fluid, and blood samples for the histological analyses were obtained intraoperatively. Arthroscopic rotator cuff repair was performed under regional or general anesthesia with the patient in the beach-chair position. Based on intraoperative evaluation, a tenotomy or tenodesis of the long head of the biceps was performed. Subacromial debridement was performed routinely with or without acromioplasty based on intraoperatively objectified bony prominence of the acromion. A biopsy of the SSP muscle was performed via a standardized method using a 5 mm–diameter Duckbill biopsy needle (8591P; Karl-Storz) medial to the myotendinous junction under visual control. Subsequently, the rotator cuff tendon-to-bone repair was performed using double-loaded 6.5-mm titanium screw-in anchors (Karl Storz SE & Co KG). Based on the intraoperative tear morphology, a single- or double-row repair was performed.

Postoperatively, the shoulders were protected in a brace at 30° of abduction for 6 weeks and passively mobilized under the control of a physical therapist. Between weeks 7 and 12, active full range of motion (ROM) exercises were allowed without strengthening. Incremental strengthening of the repaired rotator cuff muscles was started from week 13 onward.

### Assessment of Imaging Parameters

All patients underwent MRI including Dixon sequences of the shoulder (1.5-T scanner, MAGNETOM Avanto Fit; Siemens Healthcare) with a dedicated 16-channel phased-array shoulder coil. An identical protocol was used for all at the 3 different time points. The protocol consisted of 4 standard imaging planes (coronal oblique proton density weighted with fat saturation; sagittal oblique T1-weighted; sagittal oblique short tau inversion recovery; axial T2-weighted true fast imaging with steady-state free precession, 3-dimensional) and sagittal oblique 6-point Dixon sequence. To quantify intramuscular fat, fat signal fraction maps were generated on the scanner's console using the Dixon sequence.^10,24^ MRI analysis was performed by a fellowship-trained musculoskeletal radiologist (L.F.) who was blinded to clinical data (eg, pain, shoulder function) but not to the imaging at each time point.

#### Preoperative Structural Predictors

The tear size was assessed according to the Cofield classification^7^: small (<1 cm), medium (1-3 cm), large (>3-5 cm), and massive (>5 cm). SSP tendon length was measured from the myotendinous junction to tendon stump in the center of the tendon as a compromise to account for potential differences between articular-sided and bursal-sided tendon length.^12^ To measure fatty muscle infiltration, qualitative and quantitative analyses were performed. Whereas qualitative assessment involved scoring of fatty infiltration according to the Goutallier classification,^17,23^ quantitative assessment included placing freehand regions of interest on fat signal fraction maps on the most lateral slice where the scapular Y was still visible, as described previously.^20,32,54^ To evaluate SSP muscle atrophy, the tangent sign was employed.^54^

#### Tendon Integrity

Tendon continuity was assessed at 3 and 12 months postoperatively according to the Sugaya classification^46^: type 1 (repaired cuff of sufficient thickness, homogeneously low signal intensity), type 2 (sufficient thickness, partial high signal intensity area), type 3 (insufficient thickness without discontinuity), type 4 (minor discontinuity in >1 section, suggestive of a small tear), and type 5 (major discontinuity in each image, suggestive of a medium-to-large tear). The MRI examination at 12 months postoperatively determined structural integrity (Sugaya types 1, 2, and 3) or failure of structural healing (ie, retear; Sugaya types 4 and 5).

### Assessment of Clinical Outcomes

To evaluate clinical outcomes, the Constant score and subscores Pain, Activities of Daily Living (ADL), ROM, and Strength^9^ (both absolute and relative [adjusted for age and sex]), the Subjective Shoulder Value (SSV),^16^ and a shoulder satisfaction score on a scale from 1 to 4 (1 = *dissatisfied*; 2 = *rather dissatisfied*; 3 = *rather satisfied*; 4 = *highly satisfied*)^2^ were performed preoperatively as well as at 3 months (without strength measurements) and 12 months postoperatively by a trained research nurse not otherwise involved in the patients’ treatment. Given that age and sex were incorporated into the multivariate analysis, the absolute Constant score and subscores were employed as the primary clinical outcome parameter.

### Histological Assessment

After harvesting, the biopsies were immediately embedded in Cryomolds (Sakura Finetek; Sysmex Suisse AG) in Tissue-Tek OCT compound (Sakura Finetek; Sysmex Suisse AG) and frozen in –80°C 2-methylbutane (Merck) using a Snapfrost device (Excilone). The biopsies were kept in airtight 2-mL cryovials (Nalgene; VWR International) and stored at –80°C until used for further analysis.

To analyze muscle fiber composition and lipid content, 12-µm cryosections were performed perpendicular to the major fiber axes of most fiber profiles using a cryostat (CM3050S; Leica Biosystems), whereafter the sections were mounted on cryoslides and stored at –80°C until use.

The proportion of slow (MHC-I), fast (MHC-II), and hybrid-type (MHC-I/MHC-II) muscle fibers was assessed using double immunofluorescent staining for the 2 main MHC types according to a previously described method.^15^ Briefly, fixed sections were first incubated with goat serum and subsequently incubated with both primary antibodies (fast-type MHC-II antibody, No. ab91506; Abcam; slow-type MHC-I antibody, No. MAB1628; Millipore) in a bovine serum albumin–phosphate-buffered saline buffer. After thorough washing, the samples were incubated with the secondary antibodies Alexa Fluor 555–coupled anti-mouse and Alexa Fluor 488–coupled goat anti-rabbit antibody (No. A21425 and No. A11017; Thermo Fisher Life Technologies). Subsequently, the nuclei were counterstained with Hoechst 33342 (No. 62249; Thermo Fisher), after which the sections were embedded in fluorescent mounting medium (Dako; Agilent Technologies). The immunofluorescent signal was assessed by recording microscopic fields at 20-fold magnification covering the entire section in a nonoverlapping manner using an Olympus IX50 microscope with a DP72 digital camera operated by Cell Sens Dimension software (Version 1.6; Olympus). To analyze the different fiber types (slow, fast, or slow/fast), the images were imported in Photoshop CC (Adobe Systems Inc) for semiquantitative assessment. Fibers with >0.6 circularity were selected using manually-directed identification of the stained areas with the lasso tool fibers, as this indicates that they were sectioned in a perpendicular fashion. The number of slow, fast, and slow/fast hybrid fibers from the different microscopic fields were summed to reveal the total fiber population and calculate the respective mean percentages for the 3 fiber types over the entire cryosection. Per biopsy, a mean of 373 ± 189 muscle fibers were analyzed from 7 to 8 microscopic fields at 20-fold magnification using midsample sections.

The muscle lipid percentage was determined using an oil red O staining as described previously.^15^ Briefly, cryosections were thawed and fixed in 4% paraformaldehyde, stained with a working solution of oil red O (0.3 gram; VWR International) per 100 mL isopropanol (Merck) and counterstained with hematoxylin (Artechemis). Similar to the fiber type analysis, the entire stained section was recorded for analysis. The percentage of cross-sectional area representing red signal was assessed using a macro with ImageJ software (National Institutes of Health; http://rsbweb.nih.gov/ij/).

### Statistical Analysis

Differences between healed and unhealed rotator cuff groups were analyzed using the independent *t* test or Mann-Whitney *U* test for continuous variables (parametric or nonparametric data, respectively) and the chi-square test or Fisher exact test for categorical variables. Correlations between variables were analyzed using Pearson product-moment correlation coefficient (PCC) between 2 continuous variables, Kendall tau-b (KTB) correlation between ordinal and continuous variables, or point-biserial correlation (PBC) between continuous and binominal variables.

Multivariate regression analysis was used to determine the independent demographic, surgical, and structural predictors for morphological retears, as well as primary clinical outcome (12-month postoperative Constant score) and clinical improvement (change in Constant score from preoperatively to 12 months postoperatively). We input the structural parameters of the musculotendinous unit together with known independent predictors from previous research. Forward-conditional binomial multivariate logistic regression (tendon continuity, Sugaya types 1-3 vs 4-5) and stepwise multivariate linear regression (clinical outcomes and clinical improvement) were performed to analyze the independent predictors. Receiver operating characteristic (ROC) analysis with a calculation of area under the ROC curve (AUC) was performed to determine the cutoff value of SSP tendon length for structural tendon healing, Statistical analysis was performed using SPSS Statistics 25 (IBM). All statistical tests were 2-tailed, and a *P* value <.05 was considered statistically significant.

## Results

From the initially enrolled cohort (116 shoulders), 97 shoulders met the inclusion criteria for further analysis (19 patients excluded: no transmural tear intraoperatively [n = 4], surgery canceled last-minute by patient [n = 1], intraoperatively irreparable [n = 2], muscle biopsy insufficient quality [n = 2], revision operation before follow-up end [n = 1], postoperative shoulder trauma [n = 2], noncompliance during postoperative rehabilitation [n = 5], and no intraoperative biopsy performed [n = 2]). Table 1 presents preoperative demographic, clinical, surgical, and structural variables. Fatty infiltration measurements by semiquantitative analysis (Goutallier stage) showed a strong correlation with quantitative Dixon MRI measurements for the SSP (KTB, 0.72; *P* < .001), a moderate correlation for the infraspinatus (KTB, 0.65; *P* < .001), and a weak correlation for the subscapularis (KTB, 0.46; *P* < .001). Histological lipid content of the SSP showed a significant weak correlation with Goutallier stage (KTB, 0.30; *P* < .001) and Dixon MRI fat fraction (KTB, 0.27; *P* < .001).

**Table 1:** Preoperative Demographic, Clinical, Surgical, and Structural Variables^*a*^

Variable	Value
Demographic	
Age, y	58.5 ± 7.4
Sex	M: 63 (65); F: 34 (35)
Dominance	R: 90 (93); L: 7 (7)
Affected side	R: 51 (53); L: 46 (47)
Body mass index, kg/m^2^	27.1 ± 4.0
Delayed repair^*b*^	Acute: 15 (16); Chronic: 82 (84)
Clinical	
Constant score^*c*^	70.6 ± 16.9
Constant subscores	Pain: 9.4 ± 3.3; ADL: 13.7 ± 3.6; ROM: 31.3 ± 8.1; Strength^*c*^: 8.9 ± 6.6
SSV	52.1 ± 18.7
Satisfaction	1.6 ± 0.6
Surgical	
Fixation type	SR: 60 (62); DR: 37 (38)
Structural: MRI	
Tear size (Cofield)^*d*^	1: 6(6); 2: 55 (57); 3: 26 (27); 4: 10 (10)
SSP tear location	Anterior: 16 (17); Posterior: 1 (1); Both: 80 (82)
ISP tear location	Upper: 45 (46); Upper and Lower: 4 (4); None: 48 (50)
SSC tear location	Upper: 54 (56); Upper and Lower: 3 (3); None: 40 (41)
No. of tendons involved	1: 22 (23); 2: 43 (44); 3: 32 (33)
SSP tendon length, mm	30.9 ± 7.1
FI (Goutallier)	
SSP	Stage 0: 48 (50); Stage 1: 42 (43); Stage 2: 7 (7)
ISP	Stage 0: 61 (63); Stage 1: 32 (33); Stage 2: 4 (4)
SSC	Stage 0: 74 (76); Stage 1: 16 (17); Stage 2: 6 (6)
FI (Dixon MRI), %	
SSP	6.3 ± 3.9
ISP	5.9 ± 4.0
SSC	5.5 ± 3.5
Atrophy (tangent sign)	Positive: 5 (5); Negative: 92 (95)
Structural: histology	
Lipid content muscle, %	1.85 ± 3.31
Slow MHC-I, %	48.03 ± 13.42
Fast MHC-II, %	49.29 ± 12.88
Hybrid MHC-I/MHC-II, %	2.67 ± 4.11

a Values are expressed as mean ± SD or n (% within group). ADL, Activities of Daily Living; DR, double row; F, female; FI, fatty infiltration; ISP, infraspinatus; L, left; M, male; MHC, myosin heavy chain; MRI, magnetic resonance imaging; R, right; ROM, range of motion; SR, single row; SSC, subscapularis; SSP, supraspinatus; SSV, Subjective Shoulder Value.

b Delayed repair: surgery was >3 months after start of symptoms or trauma.

c Relative score (adjusted for age and sex).

d 1 = small; 2 = medium; 3 = large; 4 = massive.

### Tendon Healing

A total of 76 (78.4%) rotator cuff reconstructions were healed 1 year postoperatively. Most retears occurred in the first 3 months postoperatively, showing 17 (17.5%) MRI-confirmed retears after 3 months and 21 (21.7%) after 12 months. To include all documented early and late retears, the 12-month time point is used for further analysis. Table 2 presents an overview of the differences between healed and unhealed rotator cuff reconstructions after 12 months for the selected parameters.

**Table 2:** Comparison Between Healed and Unhealed Rotator Cuff at 12 Months Postoperatively^*a*^

Variable	Healed (n = 76; 78.4%)	Retear (n = 21; 21.7%)	*P*
Demographic			
Age, y	57.5 ± 7.3	61.9 ± 6.8	**.02**
Sex	M: 46 (60.5); F: 30 (39.5)	M: 17 (81); F: 4 (19)	.08
Delayed repair^*b*^	A: 11 (14.5); C: 65 (85.5)	A: 4 (19); C: 17 (81)	.61
Surgical			
Fixation type	SR: 43 (56.6); DR: 33 (43.4)	SR: 17 (81); DR: 4 (19)	**.04**
Structural: MRI			
Tear size (Cofield)^*c*^	2.3 ± 0.7	3 ± 0.7	**<.01**
SSP length, mm	32 ± 6.8	27 ± 7	**<.01**
FI (Goutallier)			
SSP	0.5 ± 0.6	0.9 ± 0.7	**.02**
ISP	0.4 ± 0.6	0.5 ± 0.5	.32
SSC	0.2 ± 0.5	0.5 ± 0.7	.15
FI (Dixon MRI), %			
SSP	5.8 ± 3.6	8 ± 4.5	**.03**
ISP	5.8 ± 4	6.3 ± 4.1	.66
SSC	5.5 ± 3.7	5.5 ± 2.8	.94
Tangent sign	Positive: 2 (2.6); Negative: 74 (76.3)	Positive: 3 (14.3); Negative: (85.7)	.07
Structural: histology			
Lipid content muscle, %	1.5 ± 2.4	3.3 ± 5.4	.17
Slow MHC-I, %	49.1 ± 13.5	44.1 ± 12.6	.14
Fast MHC-II, %	48.9 ± 13.1	50.7 ± 12.2	.59
Hybrid MHC-I/MHC-II, %	2 ± 3.4	5.1 ± 1.2	**.02**

a Values are expressed as mean ± SD or n (% within group). Boldface *P* values indicate statistically significant difference between groups (*P* < .05). A, acute; C, chronic; DR, double row; F, female; FI, fatty infiltration; ISP, infraspinatus; M, male; MHC, myosin heavy chain; MRI, magnetic resonance imaging; SR, single row; SSC, subscapularis; SSP, supraspinatus.

b Delayed repair: surgery was >3 months after start of symptoms or trauma.

c 1 = small; 2 = medium; 3 = large; 4 = massive.

### Predictors of Structural Healing

**Figure 1.**
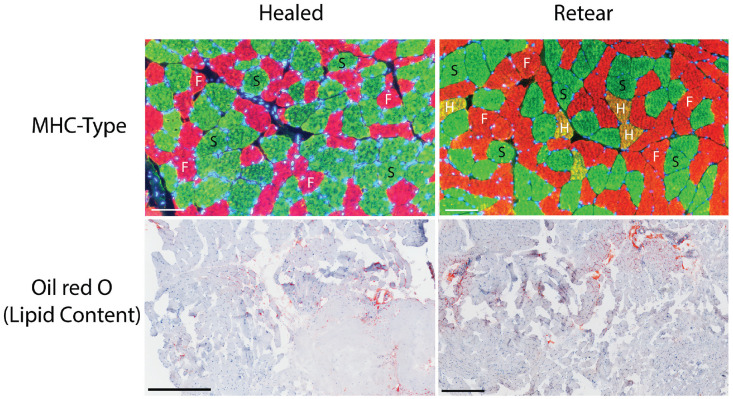
Representative histological sections of healed rotator cuff reconstruction and retear. Immunofluorescence staining for myosin heavy chain (MHC) type with slow MHC-I (S; green), fast MHC-II (F; red) and hybrid MHC-I/MHC-II (H; yellow). Oil red O staining (red) for muscle lipid content. Scale bars for MHC-type immunofluorescence represent 100 µm, and scale bars for oil red O staining represent 500 µm.

Structural predictors of the musculotendinous unit (tendon length and muscle composition) were put into the logistic multivariate model together with known independent demographic and surgical predictors or potential confounders such as sex. The model showed a high effect size, with a Cox and Snell *R*^2^; of 0.26.^8^ Older age (β = 1.12; 95% CI, 1.03-1.26; *P* = .03), shorter SSP tendon length (β = 0.89; 95% CI, 0.8-0.98; *P* = .02) and increased content of hybrid muscle fibers (β = 1.23; 95% CI, 1.07-1.42; *P* = .004) showed a significant association with failed tendon healing in the multivariate analysis (Table 3). Further analysis of these parameters using ROC analysis showed an AUC of 0.69, 0.71, and 0.70 for age, SSP tendon length, and hybrid MHC-I/MHC-II fiber type, respectively. An example of the histological sections is shown in Figure 1.

**Table 3:** Multivariate Logistic Regression of Tendon Discontinuity at 12 Months Postoperatively^*a*^

Factor	Exp β	95% CI	*P*
Demographic	—	—	
Age	1.12	1.03-1.26	**.03**
Sex	—	—	.65
Delayed repair^*b*^	—	—	.82
Surgical	—	—	
Fixation type	—	—	.32
Structural	—	—	
Tear size	—	—	.15
SSP length	0.89	0.8-0.98	**.02**
FI (Goutallier)^*c*^	—	—	
SSP	—	—	.41
ISP	—	—	.95
SSC	—	—	.79
FI (Dixon MRI)^*c*^	—	—	
SSP	—	—	.57
ISP	—	—	.76
SSC	—	—	.53
Tangent sign	—	—	.6
Histological	—	—	
Lipid content muscle^*c*^	—	—	.59
Slow MHC-I	—	—	.38
Fast MHC-II	—	—	.38
Hybrid MHC-I/MHC-II	1.23	1.07-1.42	**.004**

a Dashes indicate areas not applicable. Boldface *P* values indicate statistical significance (*P* < .05). FI, fatty infiltration; ISP, infraspinatus; MHC, myosin heavy chain; MRI, magnetic resonance imaging; SSC, subscapularis; SSP, supraspinatus.

b Delayed repair: surgery was >3 months after start of symptoms or trauma.

c Different measurement modalities of fatty infiltration were input in the regression model separately.

To analyze the interaction between tendon length and Goutallier stage of fatty infiltration on retears, the tendon length, Goutallier stage, and tendon length × Goutallier stage were put into the multivariate regression analysis. This analysis showed no significant (*P* = .76) interaction between tendon length and Goutallier stage for the SSP, infraspinatus, or subscapularis muscles. Furthermore, correlation analysis showed no significant (*P* = .67) correlation between age and hybrid MHC-I/MHC-II.

Finally, in a subgroup analysis investigating the correlation of single-row repair with structural parameters of the musculotendinous unit, only SSP fatty infiltration (Dixon MRI) showed a weak significant correlation (PBC, 0.22; *P* = .05).

### Clinical Outcomes

Healed repairs showed a significantly higher overall Constant score, SSV, and shoulder satisfaction score compared with unhealed repairs 12 months postoperatively (Table 4). Furthermore, Constant ADL and Constant ROM subscores showed significantly higher values in healed repairs. With respect to clinical improvement, only the SSV showed a significantly higher improvement of healing compared with nonhealing (Table 4).

**Table 4:** Comparison Between Clinical Score of Healed and Unhealed Rotator Cuff After 12 Months^*a*^

	Healed (n = 76; 78.4%)	Retear (n = 21; 21.7%)	*P*
Constant score (absolute)			
Pain			
12 mo postop	14.1 ± 0.2	13 ± 3.7	.06
Δ^*b*^	4.8 ± 3.8	3.5 ± 4.8	.21
ADL			
12 mo postop	19 ± 2.3	17.2 ± 4.7	**.02**
Δ^*b*^	5.2 ± 3.9	4 ± 6.8	.45
ROM			
12 mo postop	34.9 ± 4.6	31.4 ± 8.3	**.01**
Δ^*b*^	3.3 ± 8.6	1.1 ± 13.1	.48
Strength			
12 mo postop	10.6 ± 4.9	8.3 ± 4.9	.06
Δ^*b*^	2.5 ± 6.5	1.8 ± 6.5	.66
Overall			
12 mo postop	78.6 ± 11.1	70 ± 17.8	**.045**
Δ^*b*^	15.8 ± 17.7	10.4 ± 26.8	.28
Constant score (relative^*c*^)			
Overall			
12 mo postop	88.01 ± 11.9	80.3 ± 20.1	.11
Δ^*b*^	17.1 ± 18.4	11 ± 28.1	.24
SSV			
12 mo postop	87.6 ± 15.4	72.9 ± 23.4	**<.01**
Δ^*b*^	35.2 ± 22.8	21.7 ± 29.9	**.03**
Satisfaction			
12 mo postop	3.6 ± 0.7	3.1 ± 1.2	**.02**
Δ^*b*^	2.1 ± 0.9	1.7 ± 1.3	.25

a Values are expressed as mean ± SD. Boldface *P* values indicate statistically significant differences between groups (*P* < .05). ADL, Activities of Daily Living; postop, postoperatively; ROM, range of motion; SSV, Subjective Shoulder Value.

b Improvement in score from preoperatively to 12 months postoperatively.

c Adjusted for age and sex.

The 12-month postoperative Constant, SSV, and shoulder satisfaction scores showed a significant weak negative association with retear (PBC, –0.27 [*P* = .007]; –0.33 [*P* = .001]; and –0.24 [*P* = .016], respectively). Regarding all clinical scores and Constant subscores, only improvement of the SSV at 12 months postoperatively showed a significant negative correlation with retear (PBC, –0.22 [*P* = .027]).

### Predictors of Primary Clinical Outcome

The multivariate linear regression model for the primary clinical outcome (Constant score at 12 months postoperatively) showed a low effect size, with an *R*^2^; of 0.10 (Table 5).^8^ Infraspinatus fatty infiltration (Goutallier) showed a significant negative association with postoperative Constant score (β = –4.71; 95% CI, –9.30 to –0.12; *P* = .044), and fast MHC-II showed a significant positive association with postoperative Constant score (β = 0.24; 95% CI, 0.026 to 0.44; *P* = .028) (Table 5).

**Table 5:** Multivariate Linear Regression of Primary Clinical Outcome (12-Month Postoperative Constant Score)^*a*^

Factor	β (Standardized β)	95% CI	*P*
Demographic			
Age	—	—	.84
Sex	—	—	.15
Delayed repair^*b*^	—	—	.19
Surgical			
Fixation type	—	—	.12
Structural			
Tear size	—	—	.78
SSP length	—	—	.15
FI (Goutallier)^*c*^			
SSP	—		—
ISP	−4.71 (–0.20)	−9.30 to –0.12	**.044**
SSC	—	—	.26
FI (Dixon MRI)^*c*^			
SSP	—	—	.84
ISP	—	—	.26
SSC	—	—	.97
Tangent sign	—	—	.69
Histological			
Lipid content muscle^*c*^	—	—	.92
Slow MHC-I	—	—	.71
Fast MHC-II	0.24 (0.22)	0.026 to 0.44	**.028**
Hybrid MHC-I/MHC-II	—	—	.71

a Dashes indicate areas not applicable. Boldface *P* values indicate statistical significance (*P* < .05). FI, fatty infiltration; ISP, infraspinatus; MHC, myosin heavy chain; MRI, magnetic resonance imaging; SSC, subscapularis; SSP, supraspinatus.

b Delayed repair surgery was >3 months after start of symptoms or trauma.

c Different measurement modalities of fatty infiltration were input in the regression model separately.

The interactions of Goutallier fatty infiltration of the infraspinatus and fast MHC-II with clinical parameters were further analyzed. Subgroup analysis showed a significant negative correlation of infraspinatus Goutallier fatty infiltration and postoperative overall Constant score (PCC, –0.23; *P* = .022), postoperative Constant Pain (PCC, –0.2; *P* = .045) and postoperative Constant ADL (PCC, –0.24; *P* = .045). Furthermore, a significant positive correlation was observed between fast MHC-II and postoperative overall Constant score (PCC, 0.23; *P* = .023) and postoperative Constant ROM (PCC, 0.21; *P* = .043).

## Discussion

Patients with structural healing of the repair showed significantly better clinical scores (Constant, SSV, and shoulder satisfaction score) compared with those with nonhealing. However, the difference in Constant score was interpreted as not clinically relevant, as the minimal clinically important difference (MCID) of 10.4 points was higher than the observed difference of 8.6 points.^34^ On the contrary, a clinically relevant 15-point higher SSV was observed for healing compared with nonhealing (MCID = 12 points).^11^ These findings correspond with previous research, which shows improvement of both healed and unhealed tendons with slightly better clinical scores in healed patients.^26,44^ Furthermore, a stronger clinical difference between the 2 groups might be expected with longer follow-up.

This study shows the association of structural musculotendinous parameters with failed tendon healing after rotator cuff reconstruction in an arthroscopic rotator cuff reconstruction population with of Goutallier stage ≤2. Both the structural (retear) and the primary clinical outcome (Constant score) were shown to be influenced by intrinsic parameters of the musculotendinous unit. However, different predictors were observed for the structural and clinical outcome.

Decreased SSP tendon length was shown to be an independent predictor for structural rotator cuff repair failure. This corresponds with previously observed inverse correlation of tendon length and retear rate.^40^ Previous research indicates that successful repair is closely related to the elasticity of the musculotendinous unit and the ability to lateralize the unit to its intended healing site with low tension.^29^ As musculotendinous retraction is characterized by more retraction of the tendon compared with the muscle, and reduction of the defect is for 50% accounted for relengthening of the tendon,^39^ decreased tendon length may be an important parameter for increased tension of the repaired musculotendinous unit. In addition to increased tension of the musculotendinous unit, tendon shortening could be an indicator of a diminished physiological state of the tendon. Although it is widely known that tendon unloading is associated with rapid cellular, molecular, and structural changes resulting in diminished mechanical strength, the correlation of tendon length and its physiological state is unknown.^35,50^ Further investigation of the tendon length and its associated physiological state could therefore elucidate the contribution of the different components of the musculotendinous unit to healing of the rotator cuff after repair.

The fat fraction of the rotator cuff (SSP, 6.3 ± 3.9; infraspinatus, 5.9 ± 4.0; subscapularis, 5.5 ± 3.5), measured by Dixon MRI, was comparable with the literature.^32^ Similar to previous research, a significant correlation was observed between Goutallier stage and Dixon MRI fat fraction. Also, the low histological lipid content for Goutallier stage ≤2 measured in this study was comparable with a previous study investigating SSP histological morphology.^21^ A weak significant correlation (KTB, ≥0.27; *P* < .001) was observed between histological fat fraction and MRI-assessed fatty infiltration (Goutallier stage and Dixon MRI). This weak correlation might be explained by the small range of measured fat fraction in a population with Goutallier stage ≤2. A previous study showed similar small differences between histological fat fraction of patients with Goutallier stage ≤2.^21^ With respect to muscle fiber type composition, a human cadaveric SSP histological study with no macroscopic tendon pathology showed more slow-type MHC-I fibers and fewer fast-type MHC-II fibers compared with the torn SSP muscles presented in this study.^33^ An experimental study using a preclinical model has shown a similar distribution with significantly fewer slow-type MHC-I fibers in muscles with rotator cuff tears compared with intact rotator cuff muscles.^25^

In addition to tendon characteristics, increased content of hybrid muscle fibers represented an independent predictor for retear. This underlines muscular change as an important parameter for healing of the musculotendinous unit after repair. Muscular disuse is associated with various types of muscle transformation.^3,43^ This shift toward another muscle type is frequently associated with an increase in hybrid-type fibers that have altered MHC isoform expression profiles. Preclinical studies in rats showed that spinal cord transection is associated with a transition from slow MHC-I to fast MHC-IIa and MHC-IIx with a dramatic increase in slow/fast (MHC-I/MHC-IIa, MHC-IIx, MHC-IIb) hybrid muscle fibers.^47,48^ Similar results were observed in microgravity models in rats and humans with a shift from slow (MHC-I) to fast type (MHC-IIa, MHC-IIx, and MHC-IIb) and an associated substantial increase in hybrid slow/fast muscle fibers when exposed to unloading by microgravity.^13,45^ Muscle unloading by bedrest showed a significant decrease in slow MHC-I, increase in fast MHC-IIx, and an increase in overall hybrid fibers (MHC-I/MHC-IIa, MHC-IIa/ MHC-IIx, MHC-I/MHC-IIa/MHC-IIx) after 35 and 84 days of bedrest in humans.^5,19^

All in all, increase in slow/fast hybrid fibers (MHC-I/MHC-II) seems to be associated with muscular disuse atrophy and may indicate a shift from slow muscle fibers to fast muscle fibers. Previous research indicates that MHC isoforms are significantly correlated with other aspects of the muscle cell physiology, including myofibrillar proteins, metabolic enzymes, sarcoplasmic reticular proteins, and structural parameters.^37^ In an experimental preclinical model, decrease in MHC-I and MHC-IIa fibers and increase in MHC-IIb fibers was associated with a reduction in muscle fiber force production and an induction of fibrogenic, adipogenic, and autophagocytic mRNA and miRNA molecules.^25^ As this study highlights hybrid muscle fiber type content as an important independent predictor for healing of the musculotendinous unit after rotator cuff repair, further research of the associated muscle physiological state could enlighten the pathomechanism of retear. Clinically, this could have both a diagnostic and a therapeutic value. Biomarkers associated with the muscle fiber type morphology obtained by minimally invasive procedures such as synovial fluid or blood could serve as tools for a personalized surgical treatment. Furthermore, a better understanding of the pathomechanism of failure of repair could reveal potential targets for pharmacological therapies.

Although single-row repair was observed significantly more (>80%) in the retear group compared with the healed group, this parameter did not represent an independent predictor for retear. It seems that single-row repair is correlated with nonhealing of the musculotendinous unit, but underlying biological characteristics (tendon length, muscular composition) seem to influence the quality of the repair. Subgroup correlation analysis showed that from all structural parameters of the musculotendinous unit, only SSP fatty infiltration (Dixon) is significantly (PBC, 0.22; *P* = .05) correlated with single-row repair. We are therefore tempted to hypothesize that the surgical technique could be further optimized based on the biological profile of the musculotendinous unit. For example, if decreased tendon length and associated poor tendon quality prohibit the surgeon from performing a double-row repair, other techniques such as patch augmentation might be explored to reinforce the repair. A biomechanical study showed that weakened tendon structures could be fortified to nearly their original strength using patch augmentation.^1^

Linear regression showed a negative association with low effect size of infraspinatus Goutallier fatty infiltration with Constant score after 12 months. A previous study showed a similar association of preoperative infraspinatus fatty infiltration and worse clinical outcome.^22^ Apart from infraspinatus fatty infiltration, a positive association with low effect size was observed between fast MHC-II muscle fiber content and Constant score at 12 months postoperatively. Subgroup analysis correlated preoperative MHC-II muscle fiber content with increased ROM 12 months postoperatively. The association between fast MHC-II muscle fibers and muscle function and strength was also observed in previous research. For example, in elderly men it was shown that fast MHC-II–specific atrophy is predictive of muscle weakness.^49^

### Limitations

Although a cohort study design was used, study limitations should be reported. For example, the moderate sample size meant that only a limited number of predictors could be selected based on previous multivariate and meta-analyses. However, although this creates a potential bias and limited effect size for clinical outcome, we preferred this method over selecting parameters based on univariate analysis, as methodological studies showed that the latter method is associated with a high bias.^27,28^ Also, tears were repaired according to the tear pattern and anatomy. However, to control for this potential bias, the parameter “fixation type” was included in the multivariate analysis. This parameter was demonstrated to not be an independent predictor for retear in this cohort. Furthermore, although a standardized method was performed, spatial distribution could have influenced the biopsy analysis. However, with respect to muscle fiber type analysis, previous research showed robust results of fiber type characterization despite biopsy location site.^41^ Also, since the fat fraction of fatty atrophy has a known heterogeneous mediolateral distribution,^42^ the biopsies were consequently performed medial from the musculotendinous junction under visual control.

## Conclusion

The study findings confirmed that failed repairs are associated with poorer clinical outcome than healed repairs. Repair failure is more likely with older age, a shorter SSP tendon, and an increased proportion of hybrid fibers. Different intrinsic parameters showed a relevant effect on both the structural and the clinical outcome of repair. Further investigation of the relationship between the biological, structural, and functional characteristics of the musculotendinous unit might result in a more detailed understanding of the risk factors for retear and change the surgical management to increase the success rate of rotator cuff repairs.
